# Multi-Alkenylsilsesquioxanes as Comonomers and Active Species Modifiers of Metallocene Catalyst in Copolymerization with Ethylene

**DOI:** 10.3390/polym10020223

**Published:** 2018-02-24

**Authors:** Paweł Groch, Katarzyna Dziubek, Krystyna Czaja, Katarzyna Mituła, Beata Dudziec

**Affiliations:** 1 Faculty of Chemistry, Opole University, Oleska 48, 45-052 Opole, Poland; katarzyna.dziubek@uni.opole.pl (K.D.); krystyna.czaja@uni.opole.pl (K.C.); 2 Faculty of Chemistry, Adam Mickiewicz University in Poznań, Umultowska 89B, 61-614 Poznań, Poland; katarzyna.mitula@gmail.com (K.M.); beata.dudziec@gmail.com (B.D.); 3 Centre for Advanced Technologies, Adam Mickiewicz University in Poznań, Umultowska 89C, 61-614 Poznań, Poland

**Keywords:** ethylene, polyhedral oligomeric silsesquioxanes (POSS), copolymerization, metallocene, crosslinking, active site modifier

## Abstract

The copolymers of ethylene (E) with open-caged *iso*-butyl-substituted tri-alkenyl-silsesquioxanes (POSS-6-3 and POSS-10-3) and phenyl-substituted tetra-alkenyl-silsesquioxane (POSS-10-4) were synthesized by copolymerization over the ansa-metallocene catalyst. The influence of the kind of silsesquioxane and of the copolymerization conditions on the reaction performance and on the properties of the copolymers was studied. In the case of copolymerization of E/POSS-6-3, the positive comonomer effect was observed, which was associated with the influence of POSS-6-3 on transformation of the bimetallic ion pair to the active catalytic species. Functionality of silsesquioxanes and polymerization parameters affected the polyhedral oligomeric silsesquioxanes (POSS) contents in the copolymers which varied in the range of 1.33–7.43 wt %. Tri-alkenyl-silsesquioxanes were incorporated into the polymer chain as pendant groups while the tetra-alkenyl-silsesquioxane derivative could act as a cross-linking agent which was proved by the changes in the contents of unsaturated end groups, by the glass transition temperature values, and by the gel contents (up to 81.3% for E/POSS-10-4). Incorporation of multi-alkenyl-POSS into the polymer chain affected also the melting and crystallization behaviors.

## 1. Introduction

One of many advantages of polyhedral oligomeric silsesquioxanes (POSS) is their structural diversity. The POSS compounds have various structures of their silicon–oxygen cores as well as various kinds and numbers of substituents attached to them [1,2]. The POSS derivatives may be classified into three groups: mono-functional POSS, di-functional POSS, and multi-functional POSS, depending on the number of the reactive functional substituents. In the case of the (co)polymerization processes with POSS as (co)monomers, those reactions yield (co)polymers with varied architectures. The copolymers with the POSS cages as pendant side groups or end groups of the polymer chains are prepared when mono-functional POSS are used [3,4,5,6,7,8,9]. Di-functional POSS derivatives have attracted considerable attention in recent years. Depending on the types of reactive substituents, they have been successfully incorporated into such macromolecules as polyimide [9,10,11], polysiloxane [12], polyamide [13], polyurethane [14], polysulfone [15], vinylidene-arylene copolymer [16], and polyethylene [17], via the step-growth polymerization process [9,10,11,13,14,15,16,18], or as pendant groups and cross-linkage sites through the coordination copolymerization [17].

In the case of the multi-functional POSS comonomers (octa-functional POSS are mostly investigated within that group) the organic–inorganic polymers can be prepared in different ways, depending on the kinds of their reactive groups [19,20,21,22,23]. Generally, multi-functional POSS derivatives behave as nanosized cross-linkers which take part in the formation of polymer networks [19,21,22] or as precursors in the production of amphiphilic materials [23].

The multi-functional POSS derivatives are useful in preparation of (co)polymers with different structural properties. Matějka et al. [22] reported organic–inorganic epoxy networks based on diglycidyl ether of bisphenol A and poly-(oxypropylene)diamine which were reinforced with multi-functional epoxy-POSS (octa-, tetra-, and di-epoxy POSS comonomer). The obtained materials contained POSS as pendant units of the network chains or as network cross-links of various functionality. Matějka and co-workers [21] also reported modified hybrid epoxy–amine networks with mono- or multi-epoxide POSS which were produced via the reactive blending method. The POSS molecules were incorporated into the organic–inorganic networks as pendant units of the network chains or as network junctions. Liu and co-workers [20] reported the possibility of modifying epoxy resins with the use of an incompletely condensed caged POSS with three reactive hydroxyl groups (POSS-triol). The organic–inorganic hybrid polymers were prepared via in situ polymerization of POSS-triol with diglycidyl ether of bisphenol A over aluminum triacetylacetonate. Chen et al. [19] prepared a novel cross-linker, octa[(trimethoxysilyl)ethyl]-POSS, which was introduced into the hydroxyl-terminated polydimethylsiloxane matrix to prepare a series of novel silicone rubbers. The novel POSS cross-linker was found to significantly enhance the thermal stability and mechanical properties of those rubbers. The incompletely condensed and completely condensed POSS tethered with hydrophilic poly(ethylene glycol) chains were used by Yusa et al. [23] as novel organic–inorganic amphiphilic block molecules in order to obtain self-assembly nanomaterials. To the best of our knowledge, however, there are no reports available of ethylene copolymerization with multi-alkenylsilsesquioxane comonomers over the organometallic catalysts.

**Figure 1:**
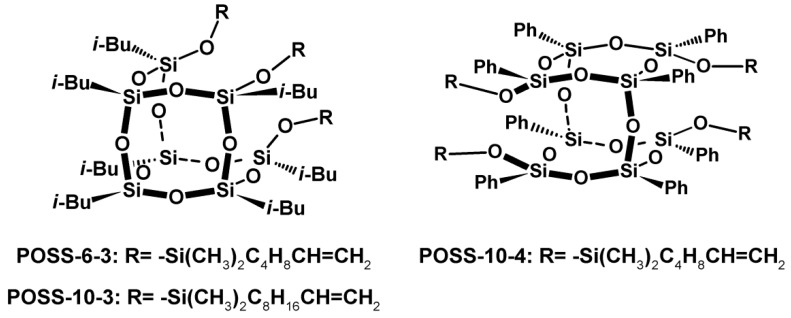
Structures of tri- and tetra-alkenyl-silsesquioxane comonomers.

Tri- and tetra-alkenyl-silsesquioxane comonomers were used in the present work for copolymerization with ethylene over the metallocene catalyst. The research involved *iso*-butyl-substituted tri-alkenyl-silsesquioxanes (POSS-6-3 and POSS-10-3) and phenyl-substituted tetra-alkenyl-silsesquioxane (POSS-10-4), which contained the incompletely condensed silicon–oxygen T_4_D_3_ and M_4_T_8_ cages, respectively (Figure 1). The POSS compounds also had various lengths of reactive alkenyl substituents in the silicon–oxygen cages (POSS-6-3 and POSS-10-3, Figure 1).

The *rac*-ethylenebis(indenyl)zirconium dichloride catalyst (*rac*-Et(Ind)_2_ZrCl_2_) activated by modified methylaluminoxane (MMAO) was applied in the (co)polymerization processes.

The aim of this work was to study the influence of the kind of POSS as well as of the polymerization conditions, such as concentration of the POSS comonomer in the feed, ethylene pressure, and reaction time, on the performance of ethylene/POSS copolymerization and on the structures and properties of the obtained copolymers. The copolymers were characterized by the following methods: nuclear magnetic resonance (^1^H and ^13^C NMR), Fourier transform infrared spectroscopy (FT-IR), differential scanning calorimetry (DSC), and gel permeation chromatography (GPC). The influence of POSS on the catalytic system activated by MMAO was investigated by ^1^H NMR. Based on the obtained results, the compositions and structural properties of copolymers were comprehensively analyzed and discussed. We also paid attention to thermal properties of copolymers, including their melting and crystallization behaviors.

## 2. Materials and Methods

### 2.1. Materials

Toluene was purchased from Chempur (Piekary Śląskie, Poland) and it was refluxed over sodium and distilled under nitrogen prior to use. Modified methylaluminoxane (MMAO, 7 wt % in toluene, Sigma-Aldrich, Saint Louis, MO, USA), *rac*-ethylenebis-(1-η^5^-indenyl)-zirconium dichloride (*rac*-Et(Ind)_2_ZrCl_2_, Sigma-Aldrich, Saint Louis, MO, USA), 1,2-dichlorobenzene-d4 (Deutero GmbH, Kastellaun, Germany), hydrochloric acid (35–38%, Chempur, Piekary Śląskie, Poland), 2,2′-metylenebis(4-methyl-6-tert-butylfenol) (Sigma-Aldrich, Saint Louis, MO, USA), and methanol (Chempur, Piekary Śląskie, Poland) were used as purchased. Toluene-d8 (Deutero GmbH, Kastellaun, Germany) was dried over molecular sieves (4 Å) and degassed in vacuo, and then it was stored and handled in vacuo.

Ethylene (Grade 3.5, Air Liquide, Kraków, Poland) and nitrogen (Messer, Chorzów, Poland) were used after passing them through a column with the sodium metal supported on Al_2_O_3_. 1,2,4-Trichlorobenzene (TCB, 99 wt %, Aldrich, Saint Louis, MO, USA) was purified by distillation.

### 2.2. Experimental Section for the Synthesis of Trialkenyl- and Tetraalkenyl-Substituted Silsesquioxanes

Tri(alkenyl)-substituted silsesquioxanes derived from cubic-type open-cage trisilanol and tetra(dec-9-enyl)-substituted double-decker silsesquioxanes were synthesized via hydrolytic condensation of incompletely condensed trisilanolisobutyl POSS (*i*-Bu_7_(Si_7_O_9_)(3OH)) and tetrasilanol silsesquioxane (DDSQ-4OH) with respective chlorosilanes. The obtained products made precursors for hydrosilylation. The procedures were based on the general methodology for alkenyl-substituted silsesquioxanes [24,25,26]. The compounds were synthesized in the Centre for Advanced Technologies, AMU (Poznań, Poland).

*i*-BuPOSS-3(OMe_2_Si-Hex) (Figure 1, POSS-6-3) was produced via hydrolytic condensation of *i*-Bu_7_(Si_7_O_9_)(3OH) with chloro(dimethyl)(hex-5-enyl)silane and it was obtained with the yield of 65%.

**^1^H NMR** (300.2 MHz, CDCl_3_, δ, ppm): 0.11 (s, 18H, –SiC***H_3_***), 0.52–0.62 (m, 20H, –C***H_2_***–(hex-5-enyl), –C***H_2_***– (*i*-Bu)), 0.95–0.97 (m, 42H, –C***H_3_*** (*i*-Bu)), 1.37–1.43 (m, 12H,–C***H_2_***- (hex-5-enyl)), 1.79–1.88 (m, 7H, –C***H***– (*i*-Bu)), 2.01–2.07 (m, 6H, –C***H_2_***– (hex-5-enyl)), 4.91–5.02 (m, 6H, ***H_2_***C=CH–), 5.74–5.88 (m, 3H, H_2_C=C***H***–). **^13^C NMR** (100.6 MHz, CDCl_3_, δ, ppm): 0.47 (***C***H_3_Si–), 18.14 (–***C***H_2_–), 22.91 (–***C***H_2_–), 23.94 (***i*-Bu**), 24.14–24.25 (***i*-Bu**), 26.02–26.19 (***i*-Bu**), 32.86 (–***C***H_2_–), 33.77 (–***C***H_2_–), 114.19 (H_2_***C***=CH–), 139.33 (H_2_C=***C***H–). **^29^Si NMR** (79.5 MHz, CDCl_3_, δ, ppm): 9.02 (–O***Si***(CH_3_)_2_–), −67.35, −67.78, −68.22.

*i*-BuPOSS-3(OMe_2_Si-Dec) (Figure 1, POSS-10-3) was produced via hydrolytic condensation of *i*-Bu_7_(Si_7_O_9_)(3OH) with chlorodimethylsilane. The product of this process, i.e., tri(hydrodimethylsilyl)-substituted silsesquioxane, was used for hydrosilylation with 1,9-decadiene to give *i*-BuPOSS-3(OMe_2_Si-Dec). The final product was obtained with the yield of 73%.

**^1^H NMR** (300.2 MHz, CDCl_3_, δ, ppm): 0.11 (s, 18H, –SiC***H_3_***), 0.54–40.56 (m, 20H, –C***H_2_***– (dec-9-enyl), –C***H_2_***– (*i*-Bu)), 0.95–0.97 (m, 42H, –C***H_3_*** (*i*-Bu)), 1.28 (m, 36H, –C***H_2_***–(dec-9-enyl)), 1.83–1.86 (m, 7H, –C***H***– (*i*-Bu)), 2.01–2.07 (m, 6H, –C***H_2_***– (dec-9-enyl)), 4.91–5.02 (m, 6H, ***H_2_***C=CH–), 5.75–5.88 (m, 3H, H_2_C=C***H***–). **^13^C NMR** (100.6 MHz, CDCl_3_, δ, ppm): 0.48 (***C***H_3_Si–), 18.32 (–***C***H_2_–), 22.64 (–***C***H_2_–) 23.38 (***i*-Bu**), 24.14–24.25 (***i*-Bu**), 26.04-26.20 (***i*-Bu**), 29.15 (–***C***H_2_–), 29.41 (–***C***H_2_–), 29.59 (–***C***H_2_–), 29.68 (–***C***H_2_–), 33.69 (–***C***H_2_–), 34.01 (–***C***H_2_–), 114.23 (H_2_***C***=CH–), 139.39 (H_2_C=***C***H–). **^29^Si NMR** (79.5 MHz, CDCl_3_, δ, ppm): 9.10 (–O***Si***(CH_3_)_2_–), −67.36, −67.83, −68.31.

DDSQ-4OSi-Dec (Figure 1, POSS-10-4) was produced via hydrolytic condensation of DDSQ-4OH with chlorodimethylsilane. The product, i.e., tetra(hydrodimethylsilyl)-substituted double-decker silsesquioxane, was used for hydrosilylation with 1,9-decadiene. The final product was obtained with the yield of 93% [25].

**^1^H NMR** (300 MHz, CDCl_3_, ppm): δ = 0.03 (s, 24H, Si(C***H_3_***)_2_-), 0.41–0.46 (m, 8H, Si-C***H_2_***-), 0.95–1.32 (m, 48H, –C***H_2_***–), 1.95–2.03 (m, 8H, –C***H_2_***–), 4.89–4.99 (m, 8H, ***H_2_***C=CH–), 5.72–5.85 (m, 4H, -CH=C***H_2_***), 7.07–7.44 (m, 40H, Ph). **^13^C NMR** (101 MHz, CDCl_3_, ppm): δ = 0.14 (Si***C***H_3_), 18.24 (–H_2_***C***–), 23.06 (–H_2_***C***–), 28.97 (–H_2_***C***–), 29.15 (–H_2_***C***–), 29.37 (–H_2_***C***–), 29.44 (–H_2_***C***–), 33.40 (–H_2_***C***–), 33.84 (–H_2_***C***–), 114.06 (H_2_***C***=CH–), 127.34–127.73 (Ph), 129.64–130.02 (Ph), 131.78 (Ph), 133.66 (Ph), 134.10–134.38 (Ph), 139.27 (H_2_C=***C***H–). **^29^Si NMR** (79.5 MHz, CDCl_3_, ppm): δ = 11.01 (–***Si***(Me)_2_–), −76.15, −78.77 (–***Si***–Ph–).

### 2.3. Procedure for Ethylene Homopolymerization and Copolymerization of Ethylene with POSS

The ethylene polymerization and ethylene/POSS copolymerization processes were carried out in a 500 cm^3^ glass reactor equipped with a propeller-like mechanical stirrer and a temperature probe. In the case of homopolymerization, the reactor was charged with 150 cm^3^ toluene and the required volume of the MMAO solution in toluene (n_Al_ = 3.01 × 10^−3^ mol). As regards copolymerization experiments, the following ingredients were used: 140 cm^3^ toluene, MMAO solution in toluene (n_Al_ = 3.01 × 10^−3^ mol), and POSS solution in 10 cm^3^ toluene ([POSS] = 0.67; 1.67; 3.33; 6.67·10^−3^ mol/dm^3^ in the reaction feed). In the next step, the solution of a metallocene catalyst in toluene (n_Zr_ = 3.5·10^−7^ mol) was introduced. Then, the reactor content was heated up to 50 °C and ethylene (0.2 or 0.5 MPa) was fed into the reaction system. The ethylene pressure and (co)polymerization temperature were maintained constant during the run. In order to terminate the polymerization process, the 5% HCl solution in methanol was introduced into the reaction feed. The obtained polymeric products were filtered and purified by stirring for 2 h with hexane (in the case of ethylene/POSS copolymers), with the 5% HCl solution in methanol, then with methanol, and then they were dried in an oven at 40 °C for 8 h.

### 2.4. Analysis of (Co)polymer, Comonomer, and Catalytic System

^1^H, ^13^C, and ^29^Si Nuclear Magnetic Resonance (NMR) analyses for POSS were performed on the Bruker UltraShield 400 and 300 MHz spectrometers (Bruker BioSpin GmbH, Rheinstetten, Germany), using CDCl_3_ as a solvent. The chemical shifts are reported in ppm with reference to the residual solvent (CHCl_3_) peaks for ^1^H and ^13^C and to TMS for ^29^Si.

The ^1^H and ^13^C NMR spectra for copolymers were recorded on the UltraShield Bruker spectrometer (400 MHz) (Bruker BioSpin GmbH, Rheinstetten, Germany) at 120 °C in 1,2-dichlorobenzene-d4 as a solvent. The ^1^H NMR spectra for the catalytic system were recorded on the same spectrometer (400 MHz) at 20 °C in toluene-d8 as a solvent, in 5 mm cylindrical glass sample tubes. All the experiments were carried out in sealed, high-vacuum systems to avoid any undesired contact with atmospheric air.

The FT-IR spectra were acquired on the Nicolet Nexus 2002 FT-IR spectrometer (Thermo Fisher Scientific, San Jose, CA, USA). The (co)polymer samples were prepared in the form of tablets which were made of the polymer powder and KBr, and their scans were taken in the range from 4000 to 400 cm^−1^ with the resolution of 2 cm^−1^. The relative POSS comonomer contents were calculated based on the ratio of the absorption band for the Si-O bond in the POSS units and the internal standard of PE. The unsaturated end groups, vinyl and trisubstituted vinylene, and their relative contents, were estimated from the peak intensities at 910 and 840–790 cm^−1^, respectively, which were normalized using the peak at 2020 cm^−1^.

The average molecular weights (*M*_w_) and molecular weight distributions (*M*_w_/*M*_n_) were found in 1,2,4-trichlorobenzene as a solvent, at 135 °C, at the flow rate of 1 cm^3^/min, on the Alliance 135 GPCV 2000 apparatus (Waters, Milford, MA, USA). The data were analyzed using the polystyrene calibration curves.

The melting temperature (*T*_m_), crystallization temperature (*T*_c_), and crystallinity degree (*X*_c_) were measured on the DSC1 calorimeter from Mettler Toledo (Columbus, OH, USA), in closed aluminum pans, under N_2_. The (co)polymer sample (10 mg) was weighed and then heated up to 170 °C at the rate of 10 °C/min, and it was kept at that temperature for 5 min to remove its thermal history, and then it was cooled down to 30 °C at 10 °C/min, and finally again heated up to 170 °C at 10 °C/min. The crystallinity degree in per cent was calculated on the basis of Δ*H*_f_ as *X*_c_ = Δ*H*_f_ × (100/290) [27].

The glass transition temperatures (*T*_g_) of the obtained polyethylene and E/POSS copolymers were studied by the differential scanning calorimetry (DSC) method using the TA Instruments DSC 2920 device (New Castle, DE, USA). All the tests were performed under nitrogen and at the rate of 10 °C/min. The *T*_g_ value was determined by the inflection point on the curve.

The gel contents of copolymers were determined by solvent extraction, according to the (Polish) standard PN-EN ISO 10147:2013-06P. The copolymer was extracted with 175 cm^3^ xylene in a Soxhlet extractor glass system. The copolymer samples (about 0.3 g) with an antioxidant (0.65 g) were extracted for 8 h. After extraction, the samples were dried in vacuum at 140 °C for 2 h to constant mass. The gel contents of the copolymer samples were calculated from the following equation: % gel content = [(weight of the sample after extraction)/(weight of the sample before extraction)] × 100%.

## 3. Results

### 3.1. Performance of E/POSS Copolymerization

**Figure 2:**
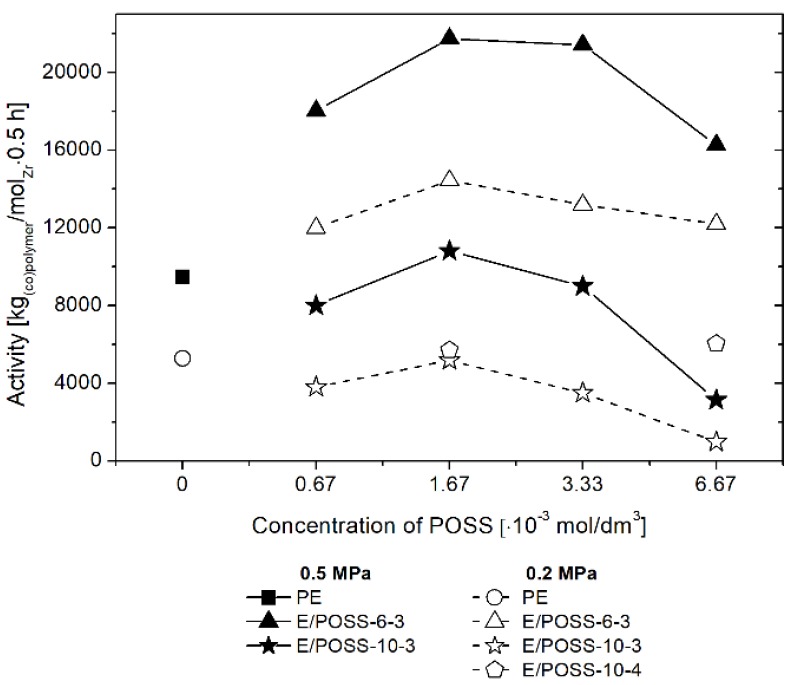
Effect of polyhedral oligomeric silsesquioxanes (POSS) comonomer on performance of ethylene copolymerization with POSS, over the *rac*-Et(Ind)_2_ZrCl_2_/MMAO catalytic system.

In the case of ethylene copolymerization with POSS, catalyzed by the *rac*-Et(Ind)_2_ZrCl_2_/MMAO catalytic system, the concentration of the POSS comonomer in the reaction feed was varied in the range from 6.67 × 10^−4^ to 6.67 × 10^−3^ mol/dm^3^ and the ethylene pressure was 0.2 or 0.5 MPa. The results of E/POSS copolymerization are presented in Figure 2.

The *rac*-Et(Ind)_2_ZrCl_2_/MMAO catalytic systems were less active in copolymerization of ethylene with POSS-10-3 and POSS-10-4 in comparison to ethylene homopolymerization (Figure 2) due to the presence of a sterically bulky POSS comonomer in the feed. In contrast, activity of the catalyst was higher for copolymerization of ethylene with the POSS-6-3 comonomer (Figure 2). It should be emphasized that this is the first example of the positive effect of a comonomer in the case of ethylene/POSS copolymerization [3,4,5,6,17]. However, a positive effect of comonomers was observed earlier for other organometallic systems, i.e., for both metallocene [28,29] and post-metallocene [30] catalysts, in the case of ethylene copolymerization with α-olefins.

The maximum activity of the metallocene catalyst in E/POSS copolymerization occurred at the POSS concentration in the feed of 1.67 × 10^−3^ mol/dm^3^, regardless of the kind of the POSS comonomer and ethylene pressure used (Figure 2). The similar relation was observed in copolymerization of ethylene with the DDSQ comonomer, as reported in our previous work [17]. The increased ethylene pressure led to higher activity of the catalyst (Figure 2).

When the results of the E/POSS-6-3 and E/POSS-10-3 copolymerization processes were compared with each other, it was found that lengthening of the reactive alkenyl substituent at the silicon–oxygen core of the POSS comonomer resulted in decreased catalytic activity (Figure 2). This could be associated with the increased steric hindrance as well as weakened interaction between the open silicon–oxygen cage of POSS-10-3 and the active center of the *rac*-Et(Ind)_2_ZrCl_2_ catalyst. The opposite relation was observed in the case of ethylene copolymerization with monoalkenyl(siloxy)- and monoalkenylsilsesquioxane which contained completely condensed *T*_8_ cages [6]. However, it should be noted that higher values of catalytic activity were obtained when the tri-alkenylsilsesquioxane comonomers were applied even at a twice lower concentration of the catalyst in the feed (up to 21,740 kg_copolymer_/mol_Zr_·0.5 h; Figure 2) in comparison with previously studied monoalkenyl(siloxy)- and monoalkenylsilsesquioxanes (3107–13,507 kg_copolymer_/mol_Zr_ × 0.5 h) [6] and the double-decker silsesquioxane derivative (8279–11,490 kg_copolymer_/mol_Zr_ × 0.5 h) [17].

**Figure 3:**
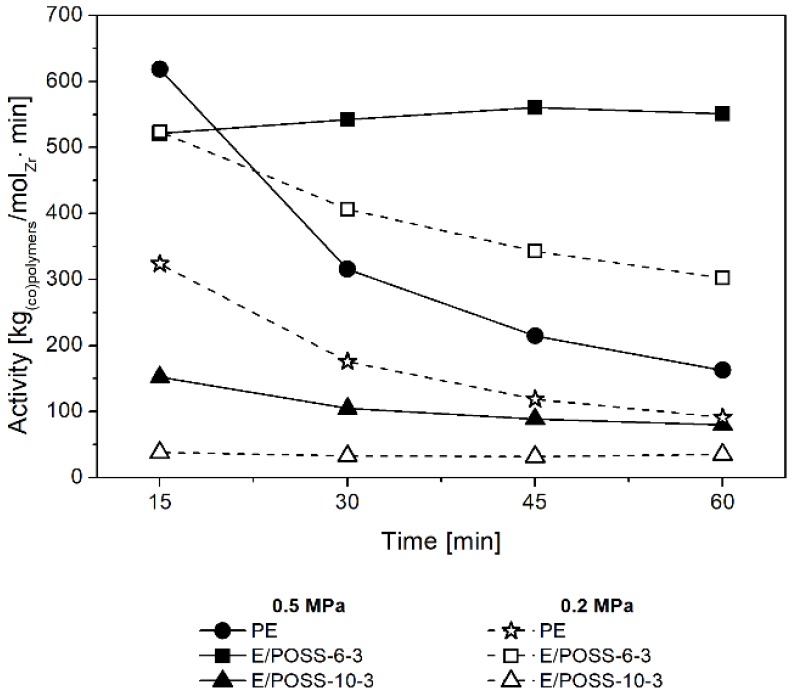
Effect of the reaction time on the activity of the *rac*-Et(Ind)_2_ZrCl_2_/MMAO catalytic system in (co)polymerization.

The effect of the reaction time on the performance of E/POSS copolymerization was also investigated (Figure 3). The activity of the ansa-metallocene/MMAO catalytic system in ethylene homopolymerization sharply decreased as the reaction time was prolonged (Figure 3).

In the case of ethylene copolymerization with POSS, the catalytic efficiency also decreased with the prolongation of time reaction, regardless of the ethylene pressure, however the changes in activity were less significant. At the same time, however, in the case of ethylene copolymerization with POSS-6-3 at p_e_ = 0.5 MPa and with POSS-10-3 at p_e_ = 0.2 MPa, the catalytic activity remained constant despite prolongation of the reaction time. This means that comonomers with incompletely condensed POSS cages do not poison the active center of the ansa-metallocene catalyst. These results indicated high stability of these active species which is attributed to the heterogeneous metallocene catalytic system, what is untypical for homogeneous analogs [31,32].

### 3.2. Influence of POSS on Ansa-Metallocene/MMAO Catalytic System

The NMR spectroscopy was successfully applied to investigate the intermolecular structure of the ionic species relevant to the catalytic homogeneous polymerization [33,34,35,36,37,38,39,40,41,42]. These literature data provided important information on the structures of intermediates formed upon the activation of metallocene with MAO in toluene. Nevertheless, the ansa-metallocene catalyst activated by MAO was studied in one case only [36].

In the case of MAO-activated metallocene, the ion pairs (**a**, **b,** and **c**) were reported on the ^1^H NMR analysis based on µ-Me resonance at –1.3, –0.85, and –0.75 ppm, respectively. A bimetallic ion pair was observed at a low Al/Zr ratio (<100 mol/mol) in which case one methyl-group acted as a bridge between Zr atoms, keeping the Me-MAO- anion in the outer coordination sphere (**a**) [33,36,37,38]. In turn, the cationic complexes [Cp_2_Zr(µ-Me)_2_AlMe_2_]^+^ Me–MAO^−^ (**b**) and Cp_2_ZrMe+ ← Me–Al≡MAO (**c**) are the major species at high Al/Zr ratios (200–4000 mol/mol) [40]. It should be noted that the complexes (**b**) and (**c**) were suggested to be the precursors of active centers for polymerization [36]. In turn, the ^1^H NMR analysis of the systems of *rac*-Et(Ind)_2_ZrCl_2_ with MAO, also at high Al/Zr ratios (300 mol/mol), showed one µ-Me signal at –0.85 ppm (detected at µ-Me signal as **b** in the case of Cp_2_ZrCl_2_/MAO) indicating the formation of the [*rac*-Et(Ind)_2_Zr(µ-Me)_2_AlMe_2_]^+^ Me–MAO^−^ complex only [36].

**Figure 4:**
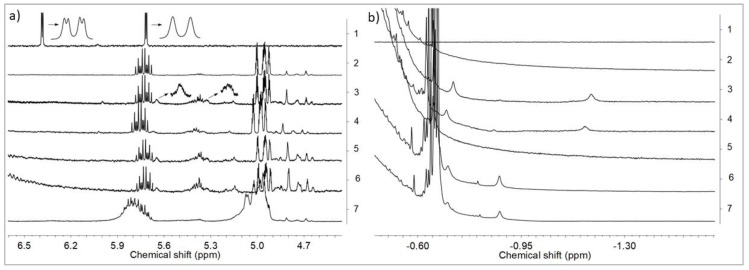
^1^H NMR spectra in two different ranges (**a**,**b**) in toluene-d8 at 20 °C of *rac*-Et(Ind)_2_ZrCl_2_ (1), MMAO (2), *rac*-Et(Ind)_2_ZrCl_2_/MMAO: Al/Zr = 50 (3), Al/Zr=100 (4), Al/Zr = 500 (5), Al/Zr = 1000 (6), and *rac*-Et(Ind)_2_ZrCl_2_/MMAO/POSS-6-3: Al/Zr = 50 (7); ([Zr] = 1.25 × 10^−2^ mol/dm^3^).

Having the above results in mind, we performed the NMR analysis of the reaction products of *rac*-Et(Ind)_2_ZrCl_2_ with MMAO, obtained in a wide range of Al/Zr molar ratios (Al/Zr = 50 to 1000 mol/mol), and of appropriate raw materials for reference. As can be seen from Figure 4, activation of *rac*-Et(Ind)_2_ZrCl_2_ by MMAO caused the down-field shift of the Ind-C_5_^1^H NMR resonance of the ansa-metallocene catalyst, irrespective of the Al/Zr molar ratios used (from 6.38 and 5.71 ppm to 5.65 and 5.33 ppm, Figure 4a: 1–6). It should be noted that the presence of three resonances at 5.73, 5.40, and from 5.00 to 4.62 ppm in MMAO (Figure 4a: 2) significantly impeded the investigation in all ^1^H NMR spectra of the catalytic systems (Figure 4a: 3–7).

Only two broadened resonances from Ind-C_5_ were found in the ^1^H NMR spectra (Figure 4a: 3) instead of the expected doublet of doublets *J*_HH_ = 3.3 and 0.7 Hz and doublet *J*_HH_ = 3.3 Hz (Figure 4a: 1). The widths of the ^1^H NMR peaks were probably affected by the exchange of the Me–MMAO^–^ counter-ions.

**Figure 5:**
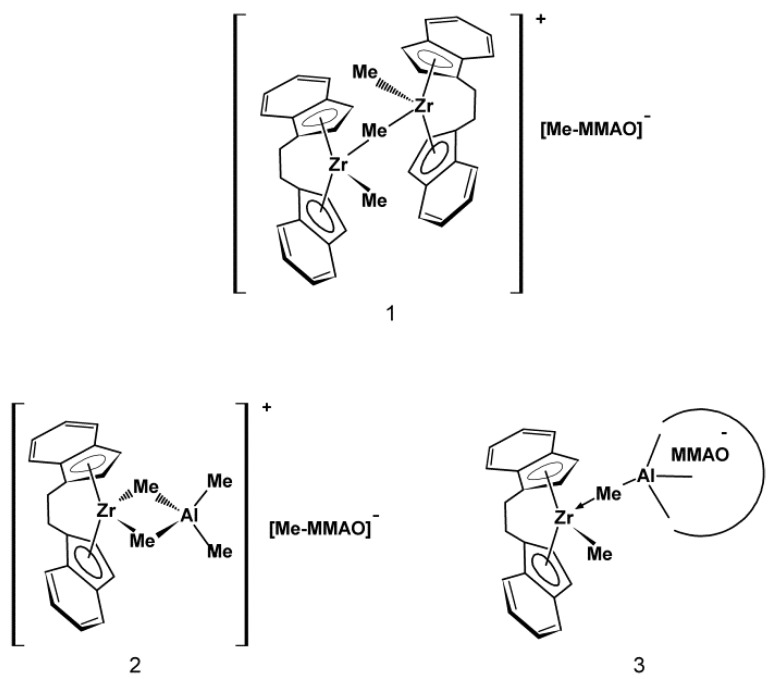
Proposed structures of catalytic intermediates formed within the *rac*-Et(Ind)_2_ZrCl_2_/MMAO catalytic system for the range of Al/Zr molar ratios used.

Moreover, the µ-Me resonances of the ion pairs at –1.2 ppm (Figure 4b: 3, 4) as well as at –0.85 ppm and –0.75 ppm were observed (Figure 4b: 3–6). Taking into account the literature data concerning the NMR analysis of metallocene/MAO systems, these signals were assigned to the structure as shown in Figure 5.

The concentrations of complexes **2** (–0.85 ppm) and **3** (–0.75 ppm) increased with the increasing Al/Zr molar ratio. In turn, the complex 1 gradually disappeared at high Al/Zr molar ratios which was confirmed by the decrease in the intensity of the µ-Me resonance at −1.2 ppm (Figure 4b: 3–6). The cationic complexes **2** and **3** turned out to be the major species at high Al/Zr ratios (500 and 1000 mol/mol) (Figure 4b: 6, 7) which was consistent with the published research reports (complexes **b** and **c**, respectively) [33,34,35,36,37,38,39,40,41,42].

It should be stressed, however, that to the best of our knowledge, the complete ansa-metallocene/MMAO/silsesquioxane system was never studied before by NMR. Meanwhile, it turned out in our research that the addition of the POSS-6-3 comonomer to the *rac*-Et(Ind)_2_ZrMe_2_/MMAO catalytic system, even at Al/Zr = 50 mol/mol, led to the disappearance of the µ-Me resonance of the complex **1** at –1.2 ppm (Figure 4 b: 7). This phenomenon is associated with the influence of the POSS comonomer on transformation of the bimetallic metallocene species **1** (inactive form) into **2** or **3** complexes (precursors of the active form). However, the observed metallocene species **2** and **3** were dominant in the catalytic system in this case, even at the low Al/Zr ratio of 50 mol/mol, which is unusual for this group of catalysts [33,34,35,36,37,38,39,40,41,42]. It is probable that the modification of the catalytic system by the POSS compound resulted in much higher activity of the catalytic system in ethylene copolymerization with the POSS-6-3 comonomer as compared to homopolymerization of ethylene (Figure 2).

Thus, the described results indicated that the POSS-6-3 compound acted not only as a comonomer but it also competed with MMAO for a bimetallic ion pair **1**. Moreover, POSS-6-3 broke the methyl bridge in complex **1** and caused the active site to be readily accessible to MMAO.

**Figure 6:**
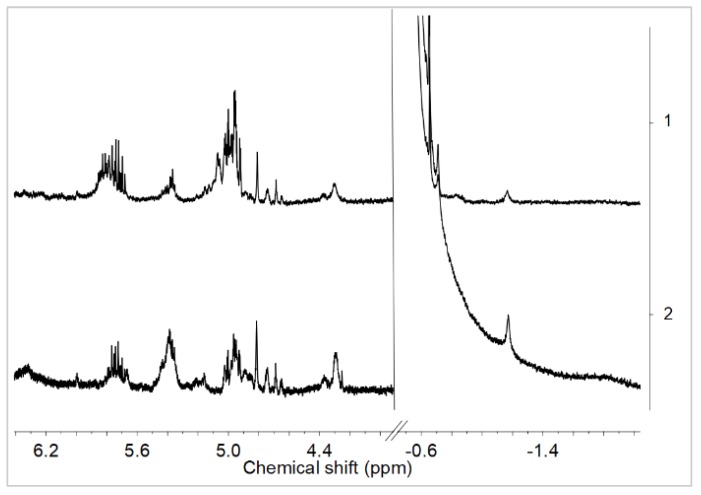
^1^H NMR spectra in toluene-d8 at 20 °C of *rac*-Et(Ind)_2_ZrCl_2_/MMAO/POSS-10-3: Al/Zr = 50 (1) and *rac*-Et(Ind)_2_ZrCl_2_/MMAO/POSS-10-4: Al/Zr = 50 (2); ([Zr] = 1.25 × 10^−2^ mol/dm^3^).

The influence of the POSS-10-3 and POSS-10-4 comonomers on the *rac*-Et(Ind)_2_ZrMe_2_/MMAO catalytic system was further investigated for the adopted Al/Zr molar ratio of 50 mol/mol. The POSS comonomers with longer alkenyl substituents were found not to affect the transformation of complex **1** into complex **2** or **3**, which was confirmed by the presence of the µ-Me resonance at –1.2 ppm in ^1^H NMR (Figure 6).

These results are consistent with the lower catalytic activity of the ansa-metallocene system in ethylene copolymerization with POSS-10-3 or POSS-10-4 in comparison to ethylene homopolymerization and especially to copolymerization of ethylene with POSS-6-3 (Figure 2).

### 3.3. Structures of E/POSS Copolymers

**Figure 7:**
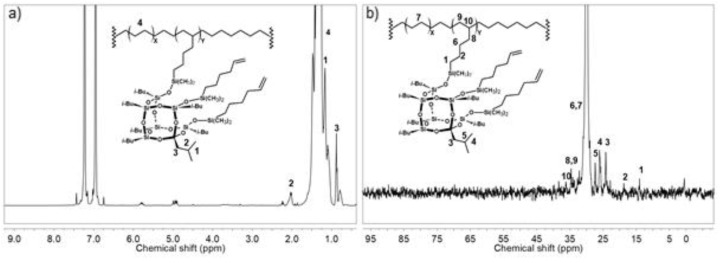
^1^H NMR (**a**) and ^13^C NMR (**b**) spectra of E/POSS-6-3 copolymer.

**Figure 8:**
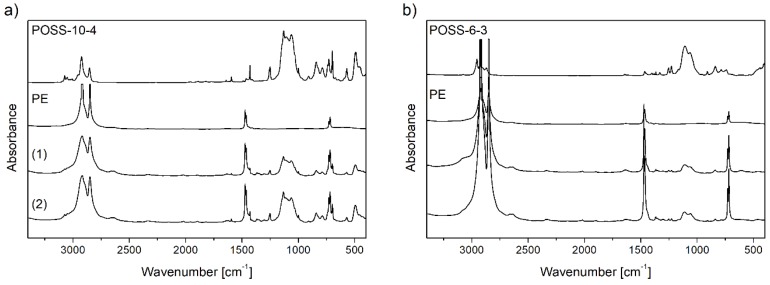
FT-IR spectra for neat POSS comonomers, polyethylenes, and E/POSS copolymers obtained over *rac*-Et(Ind)_2_ZrCl_2_ under ethylene pressure of 0.2 MPa (**a**) and 0.5 MPa (**b**) at the concentration of the POSS comonomer in the reaction feed of: 1.67 × 10^−3^ mol/dm^3^ (1) and 6.67 × 10^−3^ mol/dm^3^ (2).

The contents of POSS units incorporated into the copolymer chain (C_POSS_) and the structures of copolymers were characterized by the ^1^H and ^13^C NMR (Figure 7) and FT-IR (Figure 8) methods.

The signal pattern in the ^1^H NMR spectrum (Figure 7a) is in agreement with the general pattern for ethylene copolymers with monoalkenyl(siloxy)- or monoalkenylsilsesquioxane derivatives [4,5,6,43]. Three signals at 0.87, 1.10, and 2.03 ppm can be ascribed to the *iso*-butyl non-reactive substituent of the POSS units. A part of the (CH_2_)_x_ spacer of POSS leads to the signals in the same region of the spectrum as the polyethylene chain at about 1.32 ppm. The peaks assigned to chain unsaturation in copolymers occurred in the olefinic region (5.85–4.55 ppm). In the case of E/POSS copolymers, the ^13^C NMR spectrum (Figure 7 b) showed the signals at 14.01 ppm (C_1_ in the spacer), 18.75 ppm (C_2_ in the spacer), 29.98 ppm (C_3_ in the spacer), and 34.64 ppm (C_4_ in the spacer) for a part of the (CH_2_)_x_ spacer that were unchanged after polymerization and similar to the peaks for the *iso*-butyl group at 24.16, 26.07, and 27.35 ppm. The new peaks at 34.64 (RCH_2_–CH) and 36.21 ppm (R_3_CH) appeared due to the incorporation of POSS into the polymer chain.

A high intensity broad band with two maxima at 1130, 1055 cm^−1^ in the FT-IR spectra of neat POSS comonomers (Figure 8) indicated the presence of Si–O–Si bonds occurring in the silicon–oxygen silsesquioxane cage [44,45] and the bands at 1120, 1060 cm^−1^ were ascribed to the Si–O–Si bonds in the dimethylsiloxy spacer linking the alkenyl reactive substituent with the silicon–oxygen cage [46]. In order to determine stability of the incompletely condensed POSS cage in the polymerization conditions, the A_1120_/A_1060_ and A_1130_/A_1055_ absorbance ratios were determined using the FT-IR spectra (Figure 8) of E/POSS copolymers. The absorbance ratio was equal to about 1.09 and 1.12 for copolymers with tri- and tetra-alkenyl-POSS, respectively. Thus, it could be concluded that the structures of the POSS comonomers remained unchanged during copolymerization.

The content of the POSS comonomer incorporated into the polymer chain was found to increase with the increasing comonomer concentration in the feed (Table 1).

The copolymers with higher contents of the POSS comonomers were obtained under lower ethylene pressure (p_e_ = 0.2 MPa) during polymerization (Table 1, items 2–7 and 9–14) which was associated with a higher probability of insertion of a comonomer segment into the Zr–C bond. Interestingly, longer reaction times generally led to slightly lower incorporation of POSS into the copolymers, regardless of the reaction conditions (Table 1, items 6, 7, 10, 11, 13, 14). This result could be explained by extremely high efficiency of the catalytic system in copolymerization of ethylene with POSS, which quickly decreased the POSS concentration in the feed at the initial stage of the copolymerization process.

The structures of the POSS comonomers strongly influenced POSS incorporation into the copolymer chain and the copolymers with POSS-10-4 obtained under p_e_ = 0.2 MPa were characterized by the highest contents of the POSS units (up to 7.43 wt %) (Table 1, item 16).

Despite the presence of three and four alkenyl substituents in the POSS comonomers, the incorporation degree of the POSS comonomer into the polymer chain was unexpectedly found lower in comparison with the DDSQ comonomer under the same reaction conditions (*C*_DDSQ_ = 11.53 wt %) [17]. In the case of tri-alkenyl-POSS comonomers, in which the reactive substituents are close to each other, this phenomenon could be explained by the limited access to the active center.

For copolymers of ethylene with tri-alkenyl-POSS, the reactivity ratios of ethylene were calculated using the Fineman–Ross method [47]. It was found that the r_e_ values were equal to 162 and 182 for copolymerization of E/POSS-6-3 and E/POSS-10-3, respectively. The resulting lower reactivity of the POSS-10-3 comonomer (higher r_e_ value) could be explained by higher steric hindrance of the longer reactive group. Noteworthy is that the r_e_ values in ethylene/tri-alkenyl-POSS copolymerization were comparable to the r_e_ values for copolymerization with monoalkenyl(siloxy)- and monoalkenylsilsesquioxanes [6], but they were much higher in comparison with the DDSQ comonomer [17]. Those results confirmed that the presence of three alkenyl substituents on the same side of the POSS cage did not increase reactivity of the POSS comonomer in copolymerization with ethylene. For all the obtained polymeric products, the number–average sequence length of ethylene (n_e_) and monomer dispersity (MD) were also determined (Table 1).

The lowest values of n_e_ were observed for E/POSS-10-4 copolymers in comparison to copolymers of ethylene with POSS-6-3 and POSS-10-3 which were obtained at the same polymerization conditions (Table 2). It should be noted that the n_e_ values for ethylene copolymers with tri-alkenyl-POSS were similar to the values for copolymers which contained monoalkenyl(siloxy)- or monoalkenylsilsesquioxanes [6]. Moreover, the n_e_ values for E/POSS-10-4 copolymers were comparable to those values for E/DDSQ copolymers [17].

The monomer dispersity (MD) values were equal to about 100 irrespective of the kind of the POSS comonomer (Table 1) which suggested that the POSS units were incorporated into the polymer chain between long ethylene sequences.

**Figure 9:**
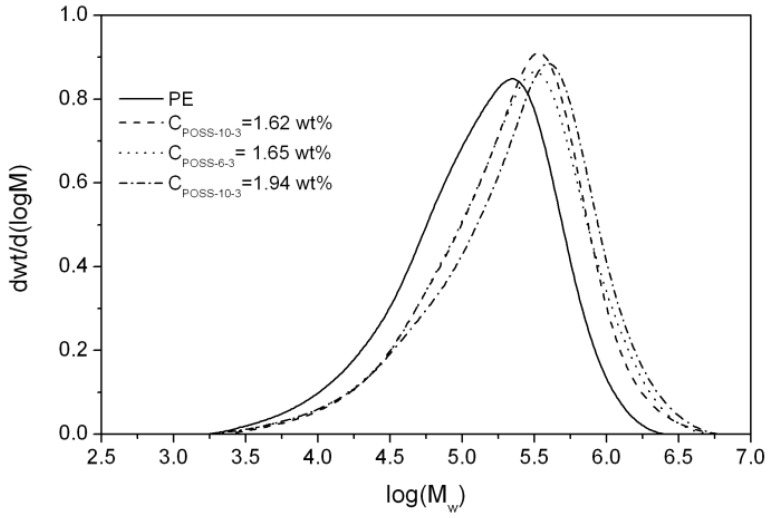
GPC curves for polyethylene and E/POSS copolymers obtained by the *rac*-Et(Ind)_2_ZrCl_2_ catalyst.

The molecular weights (*M*_w_) of the E/tri-alkenyl-POSS copolymers synthesized by *rac*-Et(Ind)_2_ZlCl_2_ were much higher in comparison to neat polyethylene, which suggested that chain termination by transfer to the monomer was significantly limited (Figure 9, Table 1). However, it should be noted that it was impossible for the E/POSS-10-4 copolymers to determine the *M*_w_ and *M*_w_/*M*_n_ values by the GPC analysis because their solubility was only partial.

Moreover, the *M*_w_ values of E/POSS increased with the increasing POSS content in the copolymer. This indicated the modifying influence of the tri-alkenyl-POSS comonomer on metallocene active sites, irrespective of the kind of reactive substituents present in the comonomer used. Based on the literature data [3,4,5,6], such phenomenon was for the first time observed in the case of ethylene copolymerization with POSS, which was catalyzed by the metallocene system. It could be explained by the interaction of incompletely condensed silicon–oxygen of the POSS comonomer with the active center of ansa-metallocene (as was previously suggested on the basis of the ^1^H NMR analysis of the complexes) or by the influence of the POSS comonomer on the formation of the ion pair, which resulted in many “open” active sites of the catalysts for the monomer insertion.

All metallocene-synthesized (co)polymers as described above were characterized by the unimodal but relatively broad molecular weight distributions (*M*_w_/*M*_n_ = 3.9–4.9) (Figure 9, Table 1). The copolymers characterized by broader molecular weight distributions were obtained when the POSS-10-3 comonomers were used in comparison to POSS-6-3, probably due to the higher POSS content in the copolymer.

**Figure 10:**
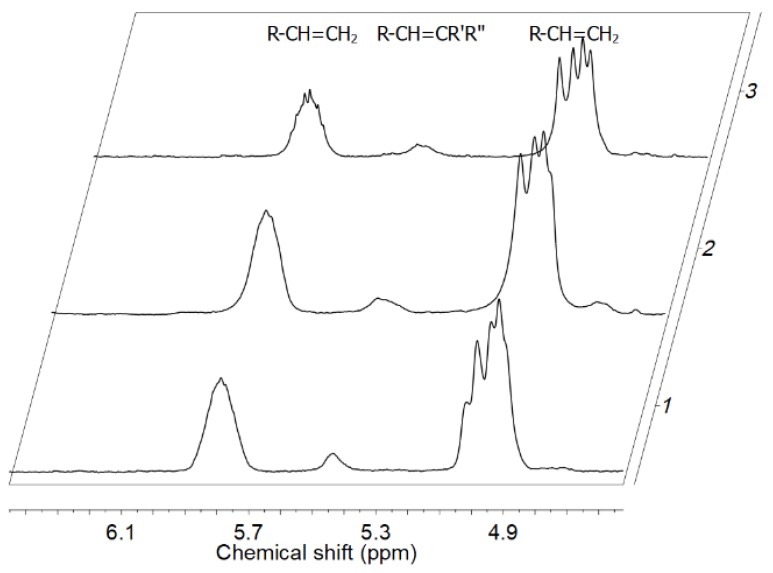
Unsaturation region in ^1^H NMR spectra of E/POSS-10-3 copolymers for various contents of POSS: 1.65 wt % (1), 1.94 wt % (2), 3.25 wt % (3).

The analysis of specific unsaturated end groups by the ^1^H NMR and FT-IR spectra for the E/POSS copolymers revealed the presence of only vinyl and tri-substituted vinylene groups (Figure 10, Table 1).

The absence of vinylidene and *trans*-vinylene end groups indicated that tri- and tetra-alkenyl-silsesquioxane did not take part in chain termination after 1,2 and 2,1-insertion of the POSS comonomer (Figure 10, Table 1) which was in line with the changes in molecular weights of the copolymers. The relative amounts of end groups increased with the increasing POSS content in the copolymers, regardless of the kind of the POSS comonomer (Table 1). The presence of trisubstituted vinylene end group in the copolymers suggested that the POSS comonomers were incorporated internally into the polymer chains [48].

Moreover, the increase in the content of vinyl end groups and increased *M*_w_ values with the increasing contents of POSS in copolymers indicated that not all alkenyl substituents of POSS took part in copolymerization. Therefore, one should consider different ways of incorporation of these multi-functional POSS units into the copolymer chains.

It is theoretically possible to obtain the macromolecules with POSS as pendant vinyl groups (type I), cyclic units (type II), and copolymers with cross-linked structures (type III) analogously to previously investigated ethylene/DDSQ copolymers [17].

**Figure 11:**
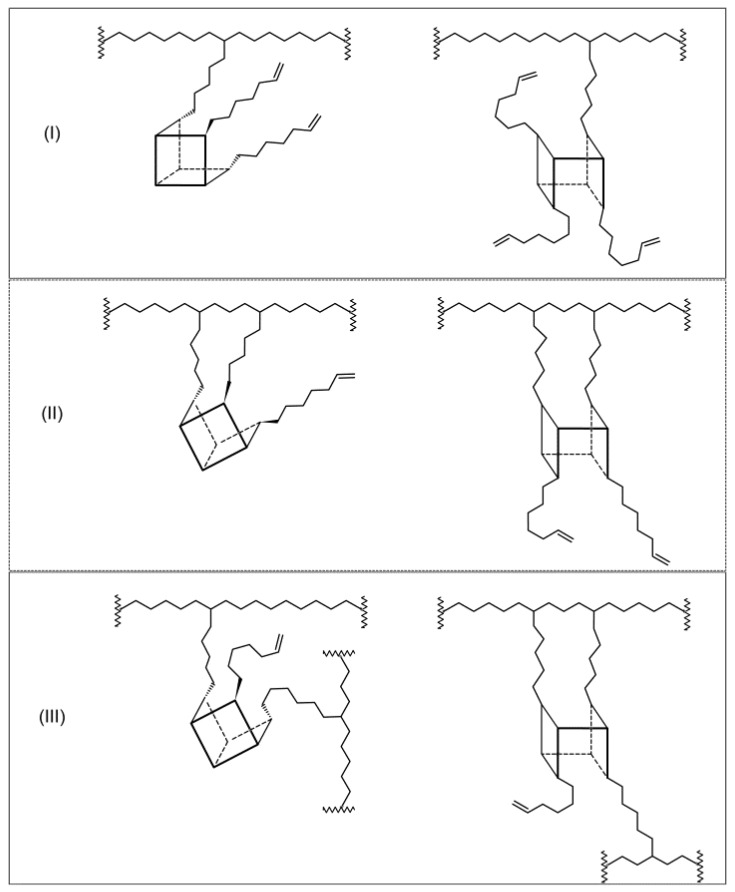
Some possible ways of incorporation of tri- or tetra-alkenyl-POSS comonomers into the polymer chain for ethylene/POSS copolymers: POSS as a pendant group (type **I**), intermolecular cyclic group (type **II**), and POSS as a cross-linking agent (type **III**).

In the case of ethylene copolymerization with the POSS comonomers which contain the reactive substituents located close to each other, the formation of cyclic units in the polymer chain could be expected (Figure 11, type II). However, the absorption band at 945 cm^−1^ associated with the cyclic structure in the main chain is not observed [49]. Noteworthy is that the relative content of vinyl groups in the case of the E/POSS-10-4 copolymer was much lower in comparison to the products with POSS-6-3 and POSS-10-3 obtained at the same reaction conditions (Table 1, items 6–11).

It is suggested that tetra-alkenyl-POSS acted as a cross-linking agent (Figure 11, type III) due to the presence of reactive alkenyl substituents on both sides of the silicon–oxygen cage, which resulted in lower steric hindrance in comparison to tri-alkenyl-POSS.

For copolymers of ethylene with tetra-alkenyl-POSS, the content of the cross-linked fraction as determined by estimation of the gel content (G) increased with the increasing content of POSS in the copolymer. It should be noted that the gel content for tetra-alkenyl-POSS-containing copolymers was higher (from 53.0% to 81.3%) in comparison to copolymers with the DDSQ comonomer (up to 65.0%) [17] despite the fact that the contents of POSS-10-4 were lower in the copolymer. This could be associated with the presence of four reactive substituents in the POSS-10-4 comonomer and hence its greater ability to create cross-linked structures.

**Figure 12:**
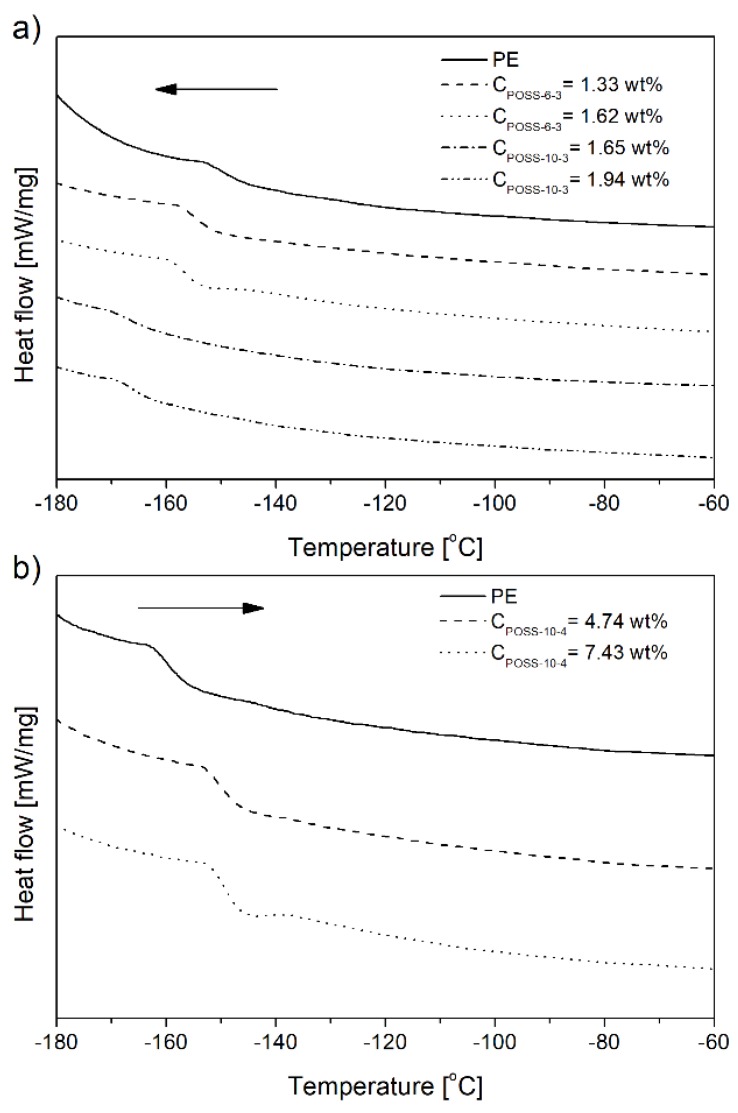
DSC curves of glass transition of selected ethylene copolymers with tri- (**a**) or tetra-alkenyl-silsesquioxane (**b**).

The incorporation of the POSS-10-4 comonomers into the polymer chain increased the *T*_g_ values significantly with the increasing POSS content (Figure 12b and Table 2) which was associated with the lower flexibility of the copolymer chain and which confirmed the cross-linked structure of the E/POSS-10-4 copolymer (Figure 11, type III).

In turn, the glass transition temperature (*T*_g_) of the ethylene copolymers with tri-alkenyl-POSS slightly decreased with the increasing contents of POSS in the copolymer (Figure 12 and Table 2). This change of the *T*_g_ value was basically in parallel with relative amounts of vinyl end groups and it indicated the presence of vinyl pendant groups.

Their increased contents in the copolymer resulted in lower *T*_g_ values (Figure 12a and Table 1 and Table 2) due to the absence of sterically rigid cyclic and cross-linked units (Figure 11, type II and III). The similar changes in *T*_g_ caused by incorporation of sterically encumbered monomer units, like cyclic rings, was reported in the literature related to ethylene copolymerization with unconjugated dienes [50].

### 3.4. Crystallization and Melting Behavior of E/POSS Copolymers

The increasing POSS content in copolymers decreased the values of the crystallinity degree (*X*_c_) and peak melting temperature (*T*_mp_), regardless of the kind of the POSS comonomer (Table 2). These results indicate that incorporation of the POSS comonomer in the copolymer resulted in disruption of regular packing of macromolecules.

**Figure 13:**
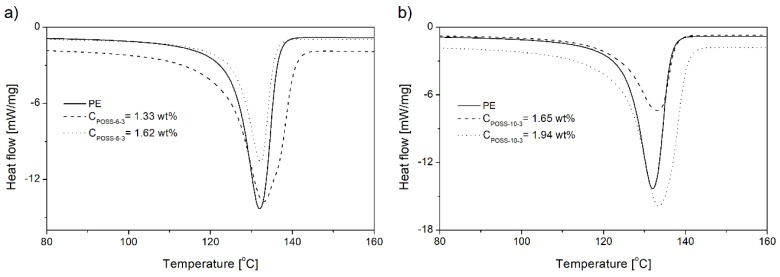
DSC curves of neat polyethylene and E/POSS copolymers synthesized by *rac*-Et(Ind)_2_ZrCl_2_ (p_e_ = 0.5 MPa) (**a**,**b**).

The endothermic peaks in the DSC curves of the copolymers were observed to get broader with the increasing POSS incorporation degree, which proved the increased heterogeneity of the copolymer, irrespective of the kind of POSS applied (Figure 13, Table 2). This phenomenon could be confirmed also by the shapes of *T*_g_ curves (Figure 12a).

The values of *T*_cp_ for the copolymers generally occurred at lower temperatures in comparison to neat polyethylene and they decreased with the increasing POSS comonomer content (Table 2). Moreover, broader exothermic peaks were observed for the copolymers produced under p_e_ = 0.2 MPa in comparison to neat polyethylene which increased with the increasing contents of the POSS units in the copolymers (Table 2, items 6–13). This phenomenon could be associated with the increased heterogeneity of the chain composition of the E/POSS copolymers. The opposite relation was observed for the E/POSS-6-3 copolymers obtained under higher ethylene pressure for which narrower exothermic peaks were visible, which suggested acceleration of the crystallization process (Table 2, items 1–5).

## 4. Conclusions

The hybrid copolymers of ethylene with open-caged *iso*-butyl-substituted tri-alkenyl-silsesquioxanes (POSS-6-3 and POSS-10-3) and phenyl-substituted tetra-alkenyl-silsesquioxane (POSS-10-4) were for the first time successfully synthesized by coordinative copolymerization with the use of the metallocene catalytic system (*rac*-Et(Ind)_2_ZrCl_2_/MMAO).

The performance of copolymerization was found to be significantly affected by the kind and concentration of the silsesquioxane comonomer in the feed as well as by the ethylene pressure and reaction time. The steric hindrance, inductive effect, and interactions between the open silicon–oxygen cage of the POSS comonomers and the active center of the metallocene catalyst significantly influenced the efficiency of polymerization. In the case of E/POSS-6-3 copolymerization, a very high catalyst activity (up to 21,740 kg_copolymer_/mol_Zr_ × 0.5 h) was noted. Moreover, the positive comonomer effect was observed for the first time in the ethylene/POSS copolymerization.

Based on the ^1^H NMR results, POSS-6-3 was found to act as a modifier of the catalytic system which caused the increased concentration of complexes **2** or **3** (precursors of active forms) at the very low Al/Zr ratio (50) which is atypical for the metallocene catalysts.

The E/POSS copolymers varied in the POSS contents, reaching up to 7.43 wt % in the case of the E/POSS-10-4 copolymer. Incorporation of the POSS comonomers into the polymer chains resulted in the increase in the molecular weights which indicated the limitation of chain termination by transfer to the monomer and suggested the modifying influence of the POSS comonomer on the metallocene active sites. Depending on the functionality of the POSS comonomer, copolymers varied in the structural architecture. Tri-alkenyl-silsesquioxanes were incorporated into the polymer chains as pendant groups. In the case of copolymers with tetra-alkenyl-silsesquioxane, the cross-linked structure was demonstrated and the gel content reached even 81.3%. These structural differences were proved by the changes in the contents of unsaturated end groups and by the glass transition temperature values. The copolymers of ethylene with multi-alkenyl-POSS were also characterized by interesting melting and crystallization behavior.

## Figures and Tables

**Table 1:** Compositions, molecular weights, and analysis of unsaturated end groups of E/POSS copolymers obtained with the use of *rac*-Et(Ind)_2_ZrCl_2_/MMAO.

Item	(Co)polymers	${\frac{A_{1120}}{A_{2020}}}^{f}$	C_POSS_^e^		n_e_^e^	MD ^e^	*M*_w_·10^3^ (g/mol) ^g^	${\frac{M_{w}}{M_{n}}}^{g}$	Analysis of Unsaturated End Groups ^f^	
			(mol %)	(wt %)					A_908_/A_2020_	A_800_/A_2020_
1	PE	0	0	0	0	0	165	1.9	0.460	0
2 ^a^	E/POSS-6-3	7.86	0.031	1.33	3226	99.969	326	3.9	0.649	0.145
3 ^b^		9.81	0.038	1.62	2632	99.962	364	4.0	0.857	0.110
4 ^b,c^		12.13	0.047	2.00	−	−	−	−	0.984	0.328
5 ^a^	E/POSS-10-3	8.83	0.034	1.65	2941	99.966	377	4.4	0.843	0.175
6 ^b^		9.98	0.040	1.94	2500	99.960	434	4.9	1.053	0.187
7 ^b,c^		8.25	0.031	1.51	−	−	526	4.8	0.769	0.541
8 ^c,d^	PE	0	0	0	0	0	226	3.9	0.291	0
9 ^a,d^	E/POSS-6-3	14.46	0.056	2.37	1786	99.944	−	−	0.945	0.161
10 ^b,d^		23.78	0.093	3.87	1075	99.907	−	−	1.664	0
11 ^b,c,d^		16.43	0.064	2.70	−	−	−	−	0.928	0.463
12 ^a,d^	E/POSS-10-3	14.66	0.057	2.74	1754	99.943	−	−	1.062	0
13 ^b,d^		17.46	0.068	3.25	1471	99.932	−	−	1.481	0.429
14 ^b,c,d^		11.07	0.043	2.08	−	−	−	−	1.337	0.309
15 ^a,d^	E/POSS-10-4	19.11	^f^ 0.075	4.74	^f^ 1333	99.925	−	−	0.565	2.663
16 ^b,c^		30.76	^f^ 0.121	7.43	^f^ 826	99.879	−	−	0.971	4.933

[POSS]: ^a^ 1.67 × 10^−3^ mol/dm^3^ and ^b^ 6.67 × 10^−3^ mol/dm^3^; *T*_r_ = 50 °C, *t*_r_ = 30, ^c^ 60 min., p_e_ = 0.5 MPa, ^d^ 0.2 MPa, ^e^ determined by ^1^H NMR, ^f^ FT-IR, ^g^ GPC.

**Table 2:** Thermal properties of PE and E/POSS copolymers synthesized over the *rac*-Et(Ind)_2_ZrCl_2_/MMAO catalytic system.

Item	(Co)polymers	*T*_g_ [°C]	*X_c_* ^c^ [%]	Melting Temperature^c^				Crystallization Temperature ^c^	
				*T*_mo_	*T*_mp_	*T*_me_	*T*_co_	*T*_cp_	*T*_ce_
1	PE ^e^	−149.7	63.2	124.8	131.0	136.4	119.7	116.5	111.9
2 ^a^	E/POSS-6-3 ^e^	−156.0	60.9	122.4	134.8	140.1	121.4	115.6	106.8
3 ^b^		−157.1	59.1	125.4	132.3	135.6	120.1	118.2	114.1
4 ^a^	E/POSS-10-3 ^e^	−166.5	58.5	122.5	134.3	138.3	120.8	115.1	108.2
5 ^b^		−166.6	60.4	124.2	133.6	140.3	120.2	116.4	107.4
6	PE ^d^	−155.0	61.2	123.2	128.6	134.6	119.8	116.5	112.8
7 ^a^	E/POSS-6-3 ^d^	−157.8	56.2	121.7	132.2	135.9	120.5	116.4	108.8
8 ^b^		−158.0	56.7	121.8	131.0	135.9	120.1	116.3	108.9
9 ^a^	E/POSS-10-3 ^d^	−157.3	54.7	119.3	129.8	133.6	119.8	115.1	108.2
10 ^b^		−162.5	50.6	122.0	129.4	135.7	118.6	116.0	108.2
11	PE ^d^	−159.3	62.2	123.2	129.6	134.6	119.8	117.5	112.8
12 ^a^	E/POSS-10-4 ^d^	−150.4	53.7	116.4	129.5	135.4	119.0	114.7	104.7
13 ^b^		−149.5	50.2	117.2	127.9	132.5	118.4	114.7	106.0

[POSS]: ^a^ 1.67 × 10^−3^ and ^b^ 6.67 × 10^−3^ mol/dm^3^, data were obtained by ^c^ DSC, p_e_ = ^d^ 0.2 and ^e^ 0.5 MPa, *T*_r_ = 50 ^o^C, *t*_r_ = 30 min.

## References

[1] Baney R.H., Itoh M., Sakakibara A., Suzuki T. (1995). Silsesquioxanes. Chem. Rev..

[2] Kickelbick G. (2007). Introduction to Hybrid Materials. Hybrid Materials.

[3] Tsuchida A., Bolln C., Sernetz F.G., Frey H., Mülhaupt R. (1997). Ethene and propene copolymers containing silsesquioxane side groups. Macromolecules.

[4] Zhang H.-X., Shin Y.-J., Yoon K.-B., Lee D.-H. (2009). Preparation and properties of propylene/POSS copolymer with rac-Et(Ind)_2_ZrCl_2_ catalyst. Eur. Polym. J..

[5] Zhang H.-X., Jung M.-S., Shin Y.-J., Yoon K.-B., Lee D.-H. (2009). Preparation and properties of ethylene/POSS copolymer with rac-Et(Ind)_2_ZrCl_2_ catalyst. J. Appl. Polym. Sci..

[6] Groch P., Dziubek K., Czaja K., Dudziec B., Marciniec B. (2017). Copolymers of ethylene with monoalkenyl- and monoalkenyl(siloxy)silsesquioxane (POSS) comonomers—Synthesis and characterization. Eur. Polym. J..

[7] Liu N., Li L., Wang L., Zheng S. (2017). Organic-inorganic polybenzoxazine copolymers with double decker silsesquioxanes in the main chains: Synthesis and thermally activated ring-opening polymerization behavior. Polymer.

[8] Hao J., Wei Y., Chen B., Mu J. (2017). Polymerization of polyhedral oligomeric silsequioxane (POSS) with perfluoro-monomers and a kinetic study. RSC Adv..

[9] Wu S., Hayakawa T., Kikuchi R., Grunzinger S.J., Kakimoto M.-A., Oikawa H. (2007). Synthesis and characterization of semiaromatic polyimides containing POSS in main chain derived from double-decker-shaped silsesquioxane. Macromolecules.

[10] Wu S., Hayakawa T., Kakimoto M.-A., Oikawa H. (2008). Synthesis and characterization of organosoluble aromatic polyimides containing POSS in main chain derived from double-decker-shaped silsesquioxane. Macromolecules.

[11] Liu N., Wei K., Wang L., Zheng S. (2016). Organic-inorganic polyimides with double decker silsesquioxane in the main chains. Polym. Chem..

[12] Miyasaka M., Fujiwara Y., Kudo H., Nishikubo T. (2010). Synthesis and characterization of hyperbranched polymer consisting of silsesquioxane derivatives. Polym. J..

[13] Wei K., Wang L., Li L., Zheng S. (2015). Synthesis and characterization of bead-like poly(*N*-isopropylacrylamide) copolymers with double decker silsesquioxane in the main chains. Polym. Chem..

[14] Huang J., Jiang P., Wen Y., Deng J., He J. (2016). Soy-castor oil based polyurethanes with octaphenylsilsesquioxanetetraol double-decker silsesquioxane in the main chains. RSC Adv..

[15] Zhang W., Xu J., Li X., Song G., Mu J. (2014). Preparation, characterization, and properties of poly(aryl ether sulfone) systems with double-decker silsesquioxane in the main chains by reactive blending. J. Polym. Sci. Part A Polym. Chem..

[16] Żak P., Dudziec B., Dutkiewicz M., Ludwiczak M., Marciniec B., Nowicki M. (2016). A new class of stereoregular vinylene-arylene copolymers with double-decker silsesquioxane in the main chain. J. Polym. Sci. Part A Polym. Chem..

[17] Groch P., Dziubek K., Czaja K., Białek M., Mituła K., Dudziec B., Marciniec B. (2018). Synthesis and structural characterization of ethylene copolymers containing double-decker silsesquioxane as pendant groups and cross-linkage sites by coordinative copolymerization. Eur. Polym. J..

[18] Liu N., Zheng S. (2016). Organic–inorganic poly(*N*-vinylpyrrolidone) copolymers with double-decker silsesquioxane in the main chains: Synthesis, glass transition, and self-assembly behavior. J. Polym. Sci. Part A Polym. Chem..

[19] Chen D., Yi S., Fang P., Zhong Y., Huang C., Wu X. (2011). Synthesis and characterization of novel room temperature vulcanized (RTV) silicone rubbers using octa[(trimethoxysilyl)ethyl]-POSS as cross-linker. React. Funct. Polym..

[20] Liu H., Zheng S., Nie K. (2005). Morphology and Thermomechanical Properties of Organic−Inorganic Hybrid Composites Involving Epoxy Resin and an Incompletely Condensed Polyhedral Oligomeric Silsesquioxane. Macromolecules.

[21] Matějka L., Strachota A., Pleštil J., Whelan P., Steinhart M., Šlouf M. (2004). Epoxy Networks Reinforced with Polyhedral Oligomeric Silsesquioxanes (POSS). Structure and Morphology. Macromolecules.

[22] Strachota A., Kroutilová I., Kovářová J., Matějka L. (2004). Epoxy Networks Reinforced with Polyhedral Oligomeric Silsesquioxanes (POSS). Thermomechanical Properties. Macromolecules.

[23] Yusa S., Ohno S., Honda T., Imoto H., Nakao Y., Naka K., Nakamura Y., Fujii S. (2016). Synthesis of silsesquioxane-based element-block amphiphiles and their self-assembly in water. RSC Adv..

[24] Mituła K., Dutkiewicz M., Dudziec B., Marciniec B., Czaja K. (2017). A library of monoalkenylsilsesquioxanes as potential comonomers for synthesis of hybrid materials. J. Therm. Anal. Calorim..

[25] Mituła K., Duszczak J., Brzakalski D., Dudziec B., Kubicki M., Marciniec B. (2017). Tetra-functional double-decker silsesquioxanes as anchors for reactive functional groups and potential synthons for hybrid materials. Chem. Commun..

[26] Mituła K., Dudziec B., Marciniec B. (2017). Synthesis of dialkenyl-substituted double-decker silsesquioxanes as precursors for linear copolymeric systems. J. Inorg. Organomet. Polym. Mater..

[27] Widman G., Riesen R. (1987). Thermal Analysis: Terms, Methods, Application.

[28] Kaminsky W., Laban A. (2001). Metallocene catalysis. Appl. Catal A Gen..

[29] Lipponen S.H., Seppälä J.V. (2011). Ethylenebis(indenyl)zirconium dichloride/methylaluminoxane-catalyzed copolymerization of ethylene and 1-alkene-n-trimethylsilanes. Organometallics.

[30] Hu P., Wang J.-Q., Wang F., Jin G.-X. (2011). Preparation, Structure, and Ethylene (Co)Polymerization Behavior of Group IV Metal Complexes with an [OSSO]-Carborane Ligand. Chem. Eur. J..

[31] Kaminsky W., Winkelbach H. (1999). Influence of supported metallocene catalysts on polymer tacticity. Top. Catal..

[32] Olabisi O., Atiqullah M., Kaminsky W. (1997). Group 4 Metallocenes: Supported and Unsupported. J. Macromol. Sci. C.

[33] Tritto I., Donetti R., Sacchi M.C., Locatelli P., Zannoni G. (1997). Dimethylzirconocene−Methylaluminoxane Catalyst for Olefin Polymerization:  NMR Study of Reaction Equilibria. Macromolecules.

[34] Lyakin O.Y., Bryliakov K.P., Semikolenova N.V., Lebedev A.Y., Voskoboynikov A.Z., Zakharov V.A., Talsi E.P. (2007). ^1^H and ^13^C NMR Studies of Cationic Intermediates Formed upon Activation of “Oscillating” Catalyst (2-PhInd)_2_ZrCl_2_ with MAO, MMAO, and AlMe_3_/[CPh_3_]^+^[B(C_6_F_5_)_4_]. Organometallics.

[35] Bryliakov K.P., Talsi E.P., Bochmann M. (2004). ^1^H and ^13^C NMR Spectroscopic Study of Titanium(IV) Species Formed by Activation of Cp_2_TiCl_2_ and [(Me_4_C_5_)SiMe_2_NtBu]TiCl_2_ with Methylaluminoxane (MAO). Organometallics.

[36] Talsi E.P., Bryliakov K.P., Semikolenova N.V., Zakharov V.A., Ystenes M., Rytterc E. (2003). ^1^H NMR characterization of intermediates formed by the activation of zirconocenes with methylaluminoxane at high Al/Zr ratios. Mendeleev Commun..

[37] Bryliakov K.P., Semikolenova N.V., Yudaev D.V., Ystenes M., Rytter E., Zakharov V.A., Talsi E.P. (2003). ^1^H and ^13^C NMR Study of the Intermediates Formed by (Cp-R)_2_ZrCl_2_ Activation with MAO and AlMe_3_/[CPh_3_][B(C_6_F_5_)_4_]. Correlation of Spectroscopic and Ethene Polymerization Data. Macromol. Chem. Phys..

[38] Bryliakov K.P., Semikolenova N.V., Yudaev D.V., Zakharov V.A., Brintzinger H.H., Ystenes M., Rytter E., Talsi E.P. (2003). ^1^H-, ^13^C-NMR and ethylene polymerization studies of zirconocene/MAO catalysts: Effect of the ligand structure on the formation of active intermediates and polymerization kinetics. J. Organomet. Chem..

[39] Babushkin D.E., Brintzinger H.-H. (2002). Activation of Dimethyl Zirconocene by Methylaluminoxane (MAO) Size Estimate for Me-MAO- Anions by Pulsed Field-Gradient NMR. J. Am. Chem. Soc..

[40] Babushkin D.E., Semikolenova N.V., Zakharov V.A., Talsi E.P. (2000). Mechanism of dimethylzirconocene activation with methylaluminoxane: NMR monitoring of intermediates at high Al/Zr ratios. Macromol. Chem. Phys..

[41] Rocchigiani L., Busico V., Pastore A., Macchioni A. (2013). Probing the interactions between all components of the catalytic pool for homogeneous olefin polymerisation by diffusion NMR spectroscopy. Dalton Trans..

[42] Ciancaleoni G., Fraldi N., Budzelaar P.H.M., Busico V., Macchioni A. (2011). Structure and Dynamics in Solution of Bis(phenoxy-amine)Zirconium Catalysts for Olefin Polymerization. Organometallics.

[43] Groch P., Dziubek K., Czaja K., Białek M., Adamczyk-Tomiak K., Rabiej S., Dudziec B. (2017). Ethylene/POSS copolymerization behavior of postmetallocene catalysts and copolymer characteristics. J. Polym. Sci. Part A Polym. Chem..

[44] Bellamy L.J. (1975). The Infrared Spectra of Complex Molecules.

[45] Anderson D.R. (1974). Analysis of Silicones.

[46] Launer P.J., Arkles B. (2013). Reprinted from Silicon Compounds: Silanes & Silicones.

[47] Fineman M., Ross S.D. (1950). Linear method for determining monomer reactivity ratios in copolymerization. J. Polym. Sci..

[48] Bruaseth I., Rytter E. (2003). Dual site ethene/1-hexene copolymerization with MAO activated (1,2,4-Me_3_Cp)_2_ZrCl_2_ and (Me_5_Cp)_2_ZrCl_2_ catalysts. Possible transfer of polymer chains between the sites. Macromolecules.

[49] Kokko E., Pietikäinen P., Koivunen J., Seppälä J.V. (2001). Long-chain-branched polyethene by the copolymerization of ethene and nonconjugated α,ω-dienes. J. Polym. Sci. Part A Polym. Chem..

[50] Song S., Wu A., Yu Y., Yang P., Fu Z., Fan Z. (2017). Nonconjugated diene homopolymerization and copolymerization with ethylene catalyzed by α-diimine Ni(II) complex/Et_2_AlCl. J. Polym. Sci. Part A Polym. Chem..

